# “We were all together”- families’ experiences of the health-promoting programme – A Healthy Generation

**DOI:** 10.1186/s12889-020-10002-1

**Published:** 2020-12-14

**Authors:** Susanne Andermo, Matthias Lidin, Mai-Lis Hellenius, Anja Nordenfelt, Gisela Nyberg

**Affiliations:** 1 Department of Global Public Health, Karolinska Institutet, Stockholm, Sweden; 2 Department of Neurobiology, Care Sciences and Society, Karolinska Institutet, Stockholm, Sweden; 3 Department of Medicine, Karolinska Institutet, Stockholm, Sweden; 4 Heart & Vascular Theme, Karolinska University Hospital, Stockholm, Sweden; 5 The Foundation A Healthy Generation, Stockholm, Sweden; 6 The Swedish School of Sport and Health Sciences (GIH), Stockholm, Sweden

**Keywords:** Family intervention, Children, Psychosocial health, Participants’ perspectives, And physical activity

## Abstract

**Background:**

Healthy lifestyle habits, including physical activity (PA), are associated with a broad range of positive psychosocial and physical health benefits. However, there are challenges involved in reaching vulnerable groups in socioeconomically disadvantaged areas. There is a lack of research on family-based PA interventions, specifically considering psychosocial health. The purpose of this study was to explore how families experienced psychosocial aspects of health after participation in a family-based programme, A Healthy Generation.

**Methods:**

A Healthy Generation is a health-promoting, family-based programme delivered in collaboration with local municipalities and sport associations in socioeconomically disadvantaged areas in Sweden. Families with children in grade 2 (8–9 years), including siblings, participate in health-promoting activities, including activity sessions, healthy meals, health information and parental support groups. Data was collected through interviews with parents and children (*n* = 23) from a controlled pilot trial of the programme. Interviews were audio recorded, transcribed and analysed using a phenomenological hermeneutical method.

**Results:**

Three themes and seven sub-themes emerged. The themes were: “*A sense of belongi*ng”, “*Awareness of one’s role as a parent*” and “*Inspiration towards new and healthier behaviours*”. In terms of *A sense of belonging*, participation in the programme was the families own free zone, where they also had the opportunity of being together with other families in the programme. For participants that were isolated and lacked a social network, their participation helped them towards social participation. During the programme, parents created an *Awareness of one’s role as a parent,* with new insights on how to act as a parent and they also negotiated differences between each other. Participation in the programme contributed to *Inspiration towards new and healthier behaviours* such as experience-based insights and healthy lifestyle changes.

**Conclusions:**

This study highlights the importance of co-participation in family-based health-promoting programmes to enhance psychosocial health among families in socioeconomically disadvantaged areas. The results give new insights into participants’ experiences of psychosocial aspects of health after participation in a family-based PA programme. This knowledge can contribute to the understanding of how to design health-promoting, family-based interventions to promote psychosocial health in socioeconomically disadvantaged areas.

**Trial registration:**

ISRCTN ISRCTN11660938. Retrospectively registered 23 September 2019.

**Supplementary Information:**

The online version contains supplementary material available at 10.1186/s12889-020-10002-1.

## Background

Healthy lifestyle habits, including regular physical activity (PA), have many physical and psychosocial health benefits for both children and adults [1–4]. Psychosocial health is a multidimensional concept, comprising of psychological and social aspects [5, 6]. Psychological aspects of health are understood in line with the term for mental health, that refers to processes of the mind, such as thinking, feeling and sensing and social aspects of health include interaction with other people, for example in a group or community [6]. Numerous reviews have demonstrated the positive impact of PA on psychosocial and mental health, including improved social interaction, mastery in the physical domain, self-perceptions, well- being, resilience and independence [2–4, 6, 7]. Among children and adolescents, level of PA has also been associated with better health-related quality of life [2]. However, few children and adults reach recommended levels for PA [8, 9]. There are also socioeconomic inequalities in health where unhealthy lifestyle habits, including lower levels of PA are more prevalent in populations with low socioeconomic status (SES) than in high SES populations [8, 10]. Children in low SES families are less likely to participate in organised sports than children in high SES families [11, 12]. Given the health benefits of PA, it is therefore important to promote PA as part of a healthy lifestyle for both children and adults, especially in socioeconomically disadvantaged areas.

Social and environmental factors, such as parents, friends, PA leaders and local communities influence PA in children. Parents, in particular, play an important role as role models for their children’s life style habits including PA, sedentary behaviour and food habits [13–15]. Different parental factors, such as logistical and financial support, co-participation and role modelling have been positively associated with increased PA in children [16]. To involve parents in health-promoting interventions has therefore been proposed as a promising solution to promote positive lifestyle changes for the whole family, as well as to increase children’s PA [17]. Previous studies investigating parents and their children’s views on family-based interventions have mostly focused on recruitment, content and delivery of the interventions [18]. Brown et al. [13] have investigated how best to recruit and retain participation in family interventions. Social, health and educational benefits were proposed as key elements for participation [13]. Jago et al. [18] have shown that enjoyment, and the opportunity to try PA, are important for recruitment, and that social aspects are important for continued participation in PA interventions. Although, some research exists on health-promoting PA interventions directed at families, few interventions have included an intervention component where parents and children exercise together. Van Slujs et al. [19] have concluded that the effect of family and community interventions remains uncertain and call for more research to further identify important key approaches. The family programme A Healthy Generation is a health-promoting programme which is directed to families with children in grade two, including siblings. The programme is currently delivered in 17 socioeconomically disadvantaged areas in 10 municipalities in Sweden. A previous pilot evaluation of the programme has shown significant intervention effects between intervention and control group on total PA and in vigorous PA during the weekends, but not in other PA measures [20]. In terms of Health Related Quality of Life (HRQOL), a previous study from the same intervention showed no significant differences between intervention and control among children or adults after the intervention [21]. There were, however, a significant improvement in HRQOL in a subgroup of children and adults with initial low HRQOL scores at baseline [21].

There is a scarcity of research on family-based PA interventions. Specifically, there is a lack of knowledge about participating families’ experiences of PA interventions in relation to psychosocial health and especially among participants in socioeconomically disadvantaged areas. To increase such understanding is crucial for the development of family-based PA interventions in order to promote health and decrease inequality in health in socioeconomically disadvantaged areas. The purpose of this study was to explore how families experienced psychosocial aspects of health after participation in a family-based programme, A Healthy Generation.

## Methods

### A healthy generation

The programme A Healthy Generation was initiated in 2011 by an eonymous, non- profit foundation, in collaboration with municipalities, local sport associations and enterprises. The programme includes four components: 1) *Activity sessions,* 2) *Healthy meals*, 3) *Health information;* and 4) *Parental support groups.* The activity sessions are conducted twice a week, one weekday and one weekend day. Different activities are offered led by a health coordinator together with a leader from local sport clubs. Examples of activities are football, basketball, dance and floorball. Most activities are held in the school’s sport hall. After each activity, a healthy, hot meal is served on weekdays and a fruit break at weekends. Discussions about healthy lifestyle habits are initiated by the health coordinators, both in activity sessions and during the healthy meals, during the different activities in the programme. Parental support groups are offered to parents four times during the programme by external coaches in each municipality. The parents attend these sessions, while their children have a regular activity session in the programme to facilitate higher attendance among the parents.

### Design

This study has a qualitative design, and draws on a phenomenological, hermeneutical approach to interpret the essential meaning of the participants´ lived experiences of psychosocial health after participation in the programme, A Healthy Generation [22, 23].

The current study is part of a controlled pilot trial that has been further described elsewhere [20, 21]. The intervention took place between August 2016 to May 2017 in 4 schools, 2 intervention- (*n* = 88) and 2 control (*n* = 83) schools, in one of the municipalities where the programme was delivered. Data for this qualitative study was collected through interviews with participants from the pilot trial about 6–8 months after the programme year. Interviews were chosen since it is a sensitive data collection method that allows the participants to reflect on their lived experiences, and for the interviewee to follow-up on what is said, how it is said and its implicit meanings [24].

### Data collection

#### Participants

A purposive sample of participants in the intervention part of the controlled pilot trial were invited to participate in interviews. The sample was based on a maximum variation in terms of age, sex, number of children, and participation in the activities during the intervention. Selected parents were first contacted by phone. Parents were informed about the study and asked if they, and their child, would like to participate. Most parents of those who were contacted agreed to participate in interviews. In total, 15 parents were contacted and 13 of them and 10 children decided to participate. Reasons for not participating were a lack of time or language skills for parents and late cancellation or that parents had declined participation for their children.

#### Interviews with parents and children

In total, 23 interviews were conducted with parents (*n* = 13) and children (*n* = 10) from the intervention group about 6 months after the programme year, see Table 1 for participant characteristics. All interviews were performed by one of the authors (SA), a female experienced qualitative researcher with training in anthropology, public health and caring science. The interviews were conducted in a place selected by the participants; either in their homes, at the children’s school or at the parents’ work. Interviews with children were conducted in their respective school or home. All parental interviews, except one interview, were conducted without the child being present in order to allow them to reflect and discuss without interruption. When children were interviewed, parents could be present if they and their child wanted, but they were advised to let the children speak for themselves. Three parents attended when their children were interviewed. During the interviews, an interview guide with semi-structured questions was used, see Appendix 1. The interview guide included questions about; the participants’ experiences from participation in the programme and the meaning in relation to their health and lifestyle. Follow-up questions, such as,” Can you tell me more/ give examples?”, were used to further explore the meaning of the participants lived experience and to encourage the ongoing narrative [25]. The interviewer tried to be sensitive to experiences that seemed to be significant for the participants. In the case of fragmented memories, especially in the interviews with children, concrete examples of activities were given to encourage the child to narrate their experiences. The interview guide was developed and discussed between the researchers, and piloted in the first interview, with the intention to include the data from the interview if the guide worked well and change the guide if necessary. The interview guide worked as intended and was not changed after the interview and the interview data was included. The interviews lasted between 30 and 70 min for parents and between 15 and 30 min for children. The interviews were digitally recorded and transcribed verbatim for analysis.

**Table 1:** Descriptive characteristics of participating adults and children

Adults	
Age, mean (SD) in years	40 (6)
Nr. of children (mean)	2.4
Female, % (count)	62 (8)
Male, % (count)	38 (5)
Born in Sweden, % (count)	54 (7)
University education, % (count)	23 (3)
Participation in the programme, mean number/total number, (SD)	30/65 (12)
Children	
Age, mean (SD) in years	9 (0.4)
Female, % (count)	80 (8)
Male, % (count)	20 (2)
Participation in the programme,mean number/total number, (SD)	37/65 (14)

### Data analysis

All data was analysed using a phenomenological-hermeneutic method [22]. At first the transcripts were read several times by one of the authors (SA) to capture the whole meaning and the first interpretation was then formulated in a naïve understanding. This process enabled an initial understanding of areas of interest in the data and their meaning. The first reading was followed by a structural analysis by the same author (SA), where meaning units were condensed by a reformulation of the essence of the text. The condensed meaning units were then sorted into sub-themes and themes. In addition, half of the transcripts were analysed by another author (ML) and sub-themes and themes were then discussed. The meaning units, condensed meaning units and themes were then reflected upon in relation to the naïve understanding, alternative themes, and possible divergent interpretations. In this sense, the meaning of the initial naïve understanding and the structural analysis was interpreted and reformulated in a circular movement between part of the text and the whole. Finally, the structural analysis was contextualised in relation to the participants’ experiences of the programme for a more comprehensive understanding. During data collection and the process of analysis, saturation of themes and sub-themes was considered to be reached. The transcripts were not returned to the participants and the participants did not provide feedback on the findings.

### Ethical considerations

The study follows the ethical principles of the Declaration of Helsinki 1964 and was approved by the Regional Ethical Review Board in Stockholm (2016/447–31/2, 2016/1254–32 and 2017/2379–32). All participants received verbal and written information about the study and parents gave informed consent in writing for themselves and their children before participation in the study. For parents, this information was first given verbally when they were invited to the interviews, and then repeated verbally and in writing before the interview. All participants were informed that participation was voluntary, about the study and how the data were handled and stored*.*

## Results

**Fig. 1:**
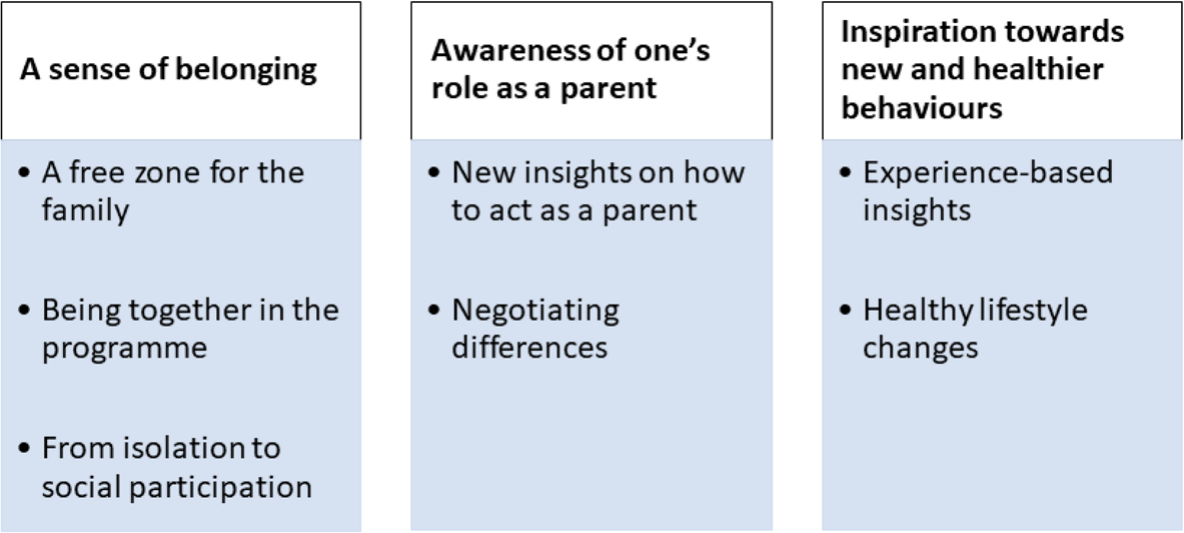
Themes and sub-themes

Three themes and seven sub-themes emerged in the structural analysis (Fig. 1). The themes were “A sense of belonging”, “Awareness of one’s role as a parent” and “Inspiration towards new and healthier behaviours”.

### A sense of belonging

#### A free zone for the family

Being together as a family during the programme activities was described as important for the participants’ family relations. Days with programme activities were the families’ own free zone, where they had the opportunity to try new activities and have fun together as a family.

For participating children, being together with their family during activities were emphasised in a joyful tone and described as fun. One child said: *“It was fun with the family and friends, and to try new activities” (child 1).* Some parents emphasised the impacts of their participation in the programme from a social perspective.

One parent said: *“We are now closer as a family, it happened when we did the activities together” (parent 6).* Other parents highlighted more practical aspects, such as the meals served on weekdays and the near-by location of activities that were perceived as facilitating factors for the families to attend. Without these meals, many parents experienced that they would not have been able to attend. They talked about how difficult it could be to have time for everything, and especially to prioritise physical activities. Participants, especially those with younger siblings, emphasised how the whole family were able to participate. To be able to have a free zone with the family, without having to rush home to do all their everyday chores:

“*It is much easier for the whole family to do something together, we'll go and everyone (in the family) is going, so that no-one has to make sure that the food is cooked.” (parent 9)*

#### Being together in the programme

Being together as a group in the programme, with other families created a sense of belonging for the participants. They had the ability to attend activities twice a week during a whole school year. At first, even though the children went to the same school, their parents described how they barely knew the other children’s parents. The continuity and the fact that whole families were able to attend the programme activities were addressed in different ways. A participant described the importance of being together in the group in relation to the programme by saying:

*“Every time we were there, we met two times each week, I felt like I was part of a group, that is why I talk about belonging, we were always together, I never felt excluded” (Parent 4)*

The duration and frequency of the programme facilitated the sense of belonging and some participants made new friendships that lasted after the programme. A child described:

*“I made a friend during A Healthy Generation, I did not have any friends in her class, but now I have become friends with her, and with one of her friends, so now I know a lot of people” (child 4)*

All participants did not make new friends after the programme, some participants described how they focused more on being with their family during the activities. However, to know more people in the neighbourhood, to say “hi” to each other at the supermarket was described as something positive and good enough for these participants.

#### From isolation to social participation

For some participants, especially those that had been feeling isolated before their participation in A Healthy Generation, expressed with emphasis how the participation helped them to break out from isolation. To participate in the activities increased social participation for them. One parent described a lonely life, where they were mostly at home. The children watched TV and the parents felt that it was difficult to get out. A Healthy Generation was described as an extra family:

*"I don't have any relatives in Sweden, but when you come here (to A Healthy Generation), it's like coming to my relatives. You eat food, you do something together". (Parent 1)*

Other participants also acknowledged the importance of the programme for social integration, especially for migrants:

*“There are many families who are trapped inside, they could come and meet each other, it is very nice with A Healthy Generation. It's a very nice start into the society, people get started.” (Parent 13)*

Social participation also included how new contacts in the neighbourhood contributed to a feeling of security. Although many parents felt that the neighbourhood was not safe for their children to play alone, the fact that more people knew their child was reassuring:

“*I know now that if he goes out by himself, there is someone who knows him. If he does something strange, someone will tell me or help him, because they know who he is. So, it feels safe that way.” (Parent 4)*

### Awareness of one’s role as parents

#### New insights on how to act as a parent

Parents described how they got new insights on how to act as parents in relation to their children’s health-related behaviours. The programme, including the parental support sessions, were important to help parents reflect about their family relations and how they acted towards their child/ children: “*We discussed how we are within the family and in relations and how to improve*…

*It was interesting, I thought differently before, the most important is how you talk with children. It is difficult sometimes, when they (the children) don’t want to do anything. I got good examples during this time, so to say” (parent 3)*

The parents also learned from each other, from other parents, children, health coordinators and local sport leaders during the programme. This way they became aware of new ways of thinking and acting towards their children. One such situation was when their children learned to eat more vegetables during the meals in the programme, as they gave each other advice.

#### Negotiating differences between parents

To participate in a health-promoting programme together with other families was also challenging, mostly due to perceived differences between the participating parents. Parents negotiated differences in relation to active participation, cultural aspects and how parents acted towards their own children. In term of active participation, parents were supposed to participate actively in the activity sessions. Even though most parents participated, some parents sat down on a bench or arrived late to the activities. Such occurrences were described as disturbing for those who participated actively. By contrast, some parents experienced that other parents participated too actively, for example when they played a sport and competed against each other. Cultural differences between parents were also brought up by a few participants, both as difficult and enriching. Difficulties mostly related to differences in parenting:

“*How one is as a parent differs from nation to nation and from person to person. It has to do with upbringing, and how you are as a person.… How should I say it, some parents don’t care about how their children behave. I had to step in as a parent, I had to tell the children, it was these things that I reacted to… it is like being a parent”. (parent 2)*

To facilitate active participation and order in the group, the health coordinator played an important role. The participants emphasised the importance of having continuity with the same health coordinator. When the coordinators knew the group, and especially the children that needed extra attention, they could take a firmer role as a leader.

### Inspiration towards new and healthier behaviours

#### Experience-based insight

Participants described how their experiences in the programme contributed to experience-based insights, how they had the opportunity to explore their own interests and how the experiences in the programme helped them to become more secure in relation to different sport activities. Even though the participants had various experience of physical activity, ranging from being very active and “sporty” as a child to very limited previous experience, to try many different sport activities within the programme was enriching. To participate together as a family in a joyful, friendly and familiar environment was important and described as a secure platform. A child that previously had been afraid to participate in physical education lessons at school described how the experiences of the programme helped her to overcome the fear and experienced more self-confidence:

*Child: All sports that I recognise, (I think that) I have done this before, that's right, I remember it from A Healthy Generation! So, when I am in school (physical education in school), I recognise it.*

*Interviewer: how does it feel then?*

*Child: Good, I feel that I recognise it. Before, I did not like physical education, because I was afraid of balls, but I love it now! (child 5)*

For the parents, it could be extra joyful to try a sport that they had participated in as a child or a new sport they had never tied:

“*I tried different sport activities that I had never tried in my life, and the children liked it too!” (parent 7)*

Skiing and ice-skating were activities that few participants, especially those born in other countries than Sweden, had previously tried. These activities were mentioned as particularly fun and exciting to learn in the programme, although difficult to begin with.

#### Healthy life-style changes

Participating in A Healthy Generation often led to healthy lifestyle changes. Some parents initially decided to participate in the programme to change their own lifestyle and others were interested because of the opportunity for their children to try different activities. Lifestyle changes included for example: to exercise more, eat healthy food and do activities together as a family. The participants were extremely grateful to the whole programme and keen to explain what it meant for them to participate. One parent commented:

“*I think it meant a lot! It helped us to think about our children’s health. Not to be unhealthy, it awoke a feeling to think about food, to think about exercise, to think healthily and to sustain this. I am very satisfied and it meant a lot to us.” (parent 10)*

Overall the experiences within the programme helped the participants to appreciate regular and more intense PA as well as healthy food habits. The participants received advice and information related to food habits from the health coordinators and they had discussions with each other, which were important reminders of how to choose healthier food patterns at home. In terms of PA, one participant described how she started to appreciate to exercise intensively during the programme:

*“You get sweaty and breathe loudly, you want to rest and sit down. They say: (the health coordinators), you need to run. You know that you need to keep running, and you want to do it, so you run.”* (parent 1)

Life-style changes also included changes in other areas in life. One parent changed job and working hours after the programme, which affected their family’s lifestyle habits starting with spending more time together, he said: *“Now, I can go home and be a father to my child”.* (parent 2).

## Discussion

The results of this study highlight the importance of co-participation in family-based health-promoting programmes to enhance psychosocial health among families in socioeconomically disadvantaged areas. Even though family- based interventions have been suggested as a promising strategy to increase children’s PA [19, 26], there is limited knowledge on participant’s experiences of the psychosocial aspects of health.

To our knowledge, this is the first study exploring how families experienced psychosocial aspects of health after participation in a family-based programme that is delivered in collaboration with local municipalities and sport associations in socio- economically disadvantaged areas.

The result of this study emphasises a range of psychosocial aspects of health from the perspective of participating families, presented in the themes: “A sense of belonging”, “An awareness of one’s roles as parents” and “Inspiration towards new and healthier behaviours”. Similar to the results in our study concerning participants’ experience of a sense of belonging and a free zone for the family, Noonan et al. [27] have previously identified that parents of 10–11 year old children viewed” Family-based time” as a beneficial factor that influenced PA intervention engagement. In their study, the included parents had a higher socioeconomic status than average, and the children participated in organised sport. The results of our study provide new insights into the psychosocial dimension of participation in family-based PA programmes in socioeconomically disadvantaged areas. Furthermore, the results of our study also suggest the potential to overcome social isolation through family-based PA programmes. Considering that both actual and perceived social isolation have been associated with an increased risk for early mortality [28] and that low social- and economic capital has been associated with poor health [29] there is an urgent need to find ways to promote psychosocial health, especially in socioeconomically disadvantaged areas. The ability to enhance a sense of belonging, including social participation through family-based interventions may therefore be of outmost importance to public health.

The result of this study also showed that participating parents achieved an awareness of their role as parents. The social dynamic and the interaction between families and leaders, despite identified challenges, were described as a means to get new insights on how to act as a parent. This is consistent with other studies about the importance of collective family activities and a sense of social connectedness with a wider family and community network for role modelling of children’s health related behaviours [30, 31], as well as research about parents’ views on parental training and the importance of group-based approach to support peer learning [32]. In contrast to the results in this study, a previously conducted review of reviews has suggested that family-based PA interventions were more effective to change PA when they were conducted in the home setting than in a community setting [19]. These results were partly explained by a higher retention rate in home-based than in community-based interventions which may facilitate increased PA. Wiltshire and Stevinson [33] have, on the other hand, addressed the importance of social relations in community-based PA interventions to increase social capital, specifically in low SES areas. This is also important considering that research has shown that families from different socioeconomic backgrounds support their children in different ways [16].

Although, some participants in this study had various sport experiences, many were unfamiliar with the different activities provided during the programme. Specifically, winter sports were new to many participants. As the results in this study show, experienced-based insights during the programme were important to get inspiration towards new and healthier behaviours. The content of the programme, and the opportunity to try different PA activities facilitated participants’ new and healthier behaviours and lifestyle changes. Furthermore, the possibility to be together with family and friends were important for participating families to feel comfortable when trying new activities. Enjoyment and social interaction have been reported in a review [34] as common reasons for participation in sport and PA. In the review, is was commented that children appreciate the chance to try different activities in a non-competitive environment with parental support [34]. Taken together, the results of our study highlight family co-participation in a community setting for psychosocial health and for promotion of healthy lifestyle changes.

### Strength and limits of the study

There are several strengths, but also limitations to this study. A strength was to focus the data collection to on one location were the programme was delivered, which provided relevant in-depth understanding on the local context and facilitated interpretation of data. In this site, the programme is well established since 2013. The programme builds on a collaboration between local municipalities, sport associations and enterprises. However, a possible limitation could also be that the result of this study may be partly dependent on contextual factors such as; the local environment, including programme facilities and leadership.

The use of interviews enabled us to capture rich data of the participants’ experiences of psychosocial health. The use of an interview guide with semi-structured questions also allowed for the participants to express their lived experiences [25] and for the interviewer to be sensitive to the areas that were brought up and experienced as meaningful by the participants. For participating parents, the period between the programme and data collection gave them enough time to reflect on the meaning of the programme for their psychosocial health. For some children, on the other hand, it is possible that it would have been better to conduct the interviews directly after or during the programme to get their immediate experiences.

Another strength of the study was the process of data analysis, with two researchers analysing and discussing the interpretation of data. Furthermore, to ensure credibility and dependability, the process of data analysis was well documented and the researchers’ pre- understanding was reflected upon to create awareness and ensure the interpretation to focus the lived experiences of the participants.

## Conclusions

This study highlights the need for family co- participation in health-promoting PA programmes to enhance psychosocial health. The findings showed how the participants created a sense of belonging, got new insights on how to act as a parent, despite some difficulties in relation to differences between participating families, and how they were able to make healthy lifestyle changes. The present study was conducted in a socioeconomically disadvantaged area. The results are relevant to the further development of family-based, health-promoting programmes to increase psychosocial health and decrease inequalities in health. Further investigation is needed on different stakeholders’ perspectives and experiences on content and delivery of the programme. Such studies could include a larger variation of intervention settings to further explore contextual opportunities and challenges**.**

## Supplementary Information

**Additional file 1.** Interview guide used in the interviews.

## Data Availability

The transcripts generated and analysed during the current study are not publicly available to maintain participant confidentiality, but the datasets used and analysed during the current study are available from the corresponding author on reasonable request.

## Abbreviations

PA: Physical activity
HRQOL: Health Related Quality of Life
SES: Socioeconomic status

## References

[1] Janssen I, Leblanc AG (2010). Systematic review of the health benefits of physical activity and fitness in school-aged children and youth. Int J Behav Nutr Phys Act.

[2] Wu XY, Han LH, Zhang JH (2017). The influence of physical activity, sedentary behavior on health-related quality of life among the general population of children and adolescents: A systematic review. PLoS One.

[3] Spruit A, Assink M, van Vugt E (2016). The effects of physical activity interventions on psychosocial outcomes in adolescents: A meta-analytic review. Clin Psychol Rev.

[4] Lubans D, Richards J, Hillman C, et al. Physical Activity for Cognitive and Mental Health in Youth: A Systematic Review of Mechanisms. Pediatrics. 2016;138 2016/08/21. 10.1542/peds.2016-1642. 10.1542/peds.2016-1642 27542849

[5] Biddle S (1995). Exercise and psychosocial health. Res Q Exerc Sport.

[6] Eime RM, Young JA, Harvey JT (2013). A systematic review of the psychological and social benefits of participation in sport for adults: informing development of a conceptual model of health through sport. Int J Behav Nutr Phys Act.

[7] Andermo S, Hallgren M, Nguyen TT (2020). School-related physical activity interventions and mental health among children: a systematic review and meta-analysis. Sports Med Open.

[8] Nyberg G (2017). Få unga rör sig tillräckligt. Centrum för idrottsforskning (ed) *De aktiva och de inaktiva: Om ungas rörelse i skola och på fritid*. Stockholm.

[9] Guthold R, Stevens GA, Riley LM (2018). Worldwide trends in insufficient physical activity from 2001 to 2016: a pooled analysis of 358 population-based surveys with 1.9 million participants. The Lancet Global health0.

[10] Love R, Adams J, Atkin A (2019). Socioeconomic and ethnic differences in children's vigorous intensity physical activity: a cross-sectional analysis of the UK Millennium Cohort Study. BMJ Open.

[11] Wijtzes AI, Jansen W, Bouthoorn SH (2014). Social inequalities in young children's sports participation and outdoor play. Int J Behav Nutr Phys Act.

[12] Blomdahl UES, Bergmark K, Lengheden L, Åkesson M (2019). Ökar ojämlikheten i föreningsidrotten? – en studie om socioekonomisk bakgrund och barns och ungdomars deltagande i idrottsförening.

[13] Brown HE, Schiff A, van Sluijs EMF. Engaging families in physical activity research: a family-based focus group study. BMC Public Health. 2015;15. 10.1186/s12889-015-2497-4. 10.1186/s12889-015-2497-4 PMC4660685 26607429

[14] Maitland C, Stratton G, Foster S (2013). A place for play? The influence of the home physical environment on children's physical activity and sedentary behaviour. Int J Behav Nutr Phys Act.

[15] Knowlden AP, Sharma M (2012). Systematic review of family and home-based interventions targeting paediatric overweight and obesity. Obes Rev.

[16] Brockman R, Jago R, Fox KR (2009). “Get off the sofa and go and play”: family and socioeconomic influences on the physical activity of 10–11 year old children. BMC Public Health.

[17] Marsh S, Foley LS, Wilks DC (2014). Family-based interventions for reducing sedentary time in youth: a systematic review of randomized controlled trials. Obes Rev.

[18] Jago R, Davis L, McNeill J (2011). Adolescent girls' and parents' views on recruiting and retaining girls into an after-school dance intervention: implications for extra-curricular physical activity provision. Int J Behav Nutr Phys Act.

[19] van Sluijs EM, Kriemler S, McMinn AM (2011). The effect of community and family interventions on young people's physical activity levels: a review of reviews and updated systematic review. Br J Sports Med.

[20] Nyberg G, Andermo S, Nordenfelt A, et al. Effectiveness of a Family Intervention to Increase Physical Activity in Disadvantaged Areas-A Healthy Generation, a Controlled Pilot Study. Int J Environ Res Public Health. 2020;17 2020/05/31. 10.3390/ijerph17113794. 10.3390/ijerph17113794 PMC7312597 32471080

[21] Andermo S, Hellénius ML, Lidin M (2020). Effectiveness of a family intervention on health-related quality of life-a healthy generation, a controlled pilot trial. BMC Public Health.

[22] Lindseth A, Norberg A (2004). A phenomenological hermeneutical method for researching lived experience. Scand J Caring Sci.

[23] Patton MQ. Qualitative evaluation and research methods. Fourth ed. Thousand Oaks: SAGE Publications, Inc; 2015.

[24] Kvale S (2007). Doing interviews.

[25] Van Manen M (1997). Researching lived experience : human science for an action sensitive pedagogy.

[26] O'Connor TM, Jago R, Baranowski T (2009). Engaging parents to increase youth physical activity a systematic review. Am J Prev Med.

[27] Noonan RJ, Boddy LM, Fairclough SJ (2017). Parental perceptions on Childrens' out-of-school physical activity and family-based physical activity. Early Child Dev Care.

[28] Holt-Lunstad J, Smith TB, Baker M (2015). Loneliness and social isolation as risk factors for mortality: a meta-analytic review. Perspect Psychol Sci.

[29] Ahnquist J, Wamala SP, Lindstrom M (2012). Social determinants of health--a question of social or economic capital? Interaction effects of socioeconomic factors on health outcomes. Soc Sci Med.

[30] Ickes S, Mahoney E, Roberts A (2016). Parental Involvement in a School-Based Child Physical Activity and Nutrition Program in Southeastern United States: A Qualitative Analysis of Parenting Capacities. Health Promotion Pract.

[31] Dwyer GM, Higgs J, Hardy LL (2008). What do parents and preschool staff tell us about young children's physical activity: a qualitative study. Int J Behav Nutr Phys Act.

[32] Jago R, Steeds JK, Bentley GF (2012). Designing a physical activity parenting course: parental views on recruitment, content and delivery. BMC Public Health.

[33] Wiltshire G, Stevinson C (2018). Exploring the role of social capital in community-based physical activity: qualitative insights from parkrun. Qualitative Res Sport Exercise Health.

[34] Allender S, Cowburn G, Foster C (2006). Understanding participation in sport and physical activity among children and adults: a review of qualitative studies. Health Educ Res.

