# Supplementary material for: “We were all together”- families’ experiences of the health-promoting programme – A Healthy Generation
Source: BMC Public Health. 2020 Dec 14;20:1911. doi: 10.1186/s12889-020-10002-1 (PMC7737279; doi:10.1186/s12889-020-10002-1)
Supplement: Supplementary file 1 — Additional file 1. Interview guide used in the interviews. [file 12889_2020_10002_MOESM1_ESM.docx]

## Interview guide- parents

1. How did you come into contact with A Healthy Generation?
   1. What did you expect initially, when you decided to participate in A Healthy Generation?
      - Did you have any hopes, which ones?
      - Did you have any concerns, which ones?
   2. Can you tell me about your family's participation in A Healthy Generation?
      - Who in the family participated, how often?
      - How did you experience to participate together with other families (both children and parents)?
   3. How did you experience the activities in A Healthy Generation? (activities (activity sessions, meals, health information and parental support groups)
      - What activities did you enjoy or did not enjoy, can you give examples?
      - What experiences do you have with the meals?
   4. What has it meant for your family to participate in A Healthy Generation?
   5. Can you describe your relations to the other families that participated in A Healthy Generation (before, during and after)?
   6. How did you experience the contact with the other people you came into contact with in A Healthy Generation?
   7. Can you describe if you have made any life style changes during or after your participation in the programme?
      - If so, can you describe these changes and the process of change?
   8. Is there anything else you want to tell me?
   9. How have you experienced this conversation?

Thank you for your participation!

## Interview guide- children

1. Have you been participating in A Healthy Generation?
2. Who participated together with you in A Healthy Generation? (parents, siblings?)
3. What did you do when you were in A Healthy Generation?
4. Can you tell me about what activities (sports, outdoor activities) you got to try while you were in A Healthy Generation?
   - - Were there any activities you enjoyed? (which activities? What was good about these?)
     - Were there any activities you didn't like? (which activities? what didn't you like about them?)
     - Were there any activities that you found specifically difficult or easy? (which ones?)
5. Did you eat together with the other children and their parents afterwards (after the activity sessions)?
   - - What was it like to eat together?
     - What did you think about the food in A Healthy Generation?
     - What do you usually eat at home?
6. Did you know any children who were in A Healthy Generation since before? (how did you know them?)
   - - How was it like to participate in A Healthy Generation with other children from your school (any new friends, other relational issues)?
7. You told me before about your family’s participation, how was it for you that they were there?
   - - Did you know the other children's parents since before?
     - What was it like to participate with other families?
8. Now, after A Healthy Generation, what do you usually do after school and in the weekends?
9. Is there anything else you want to tell me?
10. How have you experienced this conversation?

Thank you for your participation!
